# Epidermolysis Bullosa: A Case of Successful Total Hip Arthroplasty

**DOI:** 10.7759/cureus.7508

**Published:** 2020-04-02

**Authors:** Sanad H Kawasmi, Jihad Ajlouni, Qusai Almanaseer, Laith Kaylani, Abbas Hassan

**Affiliations:** 1 Orthopaedics, University of Jordan, Amman, JOR; 2 Orthopaedics, Jordan University Hospital, Amman, JOR; 3 Plastic and Reconstructive Surgery, Northwestern University Feinberg School of Medicine, Chicago, USA

**Keywords:** epidermolysis bullosa, total hip arthroplasty, femoral head necrosis, case report

## Abstract

Epidermolysis bullosa (EB) is a rare dermatological disease in which patients suffer from skin fragility and blisters. One of the major complications is the development of skin infections, which may preclude surgical intervention. We present a case of a 49-year-old female with a past medical history of EB, who presented to the emergency department (ED) with right groin pain of one-hour duration after falling on her right side. The patient underwent a successful open reduction and internal fixation for her right hip without complications. Over the course of three months after the procedure, she experienced worsening of the pain accompanied by skin necrosis and total collapse of the femoral head. Subsequent total hip replacement surgery was performed using a cementless (Zimmer, Warsaw, IN) prosthesis and fixated via cannulated screws. To decrease the risk of infection, IV cefazolin was given as a prophylactic antibiotic preoperatively. Vancomycin IV and imipenem/cilastatin IV were given for four days postoperatively. We made sure that our patient is experiencing the least possible pain by giving sufficient analgesics after the surgery. We used morphine, paracetamol, and gabapentin for pain control. For 25 days after the surgery, the patient did not complain of any pain. Upon follow-up, sutures were removed, and no surgical wound infection, rashes, or lacerations were noted. We encourage orthopedic surgeons dealing with patients suffering from dermatological conditions with fragile skin such as EB and decreased level of activity that requires total hip arthroplasty to proceed with the surgical intervention after considering adequate infection control to improve quality of life.

## Introduction

Epidermolysis bullosa (EB) is a rare dermatological disease in which patients suffer from skin fragility and blisters. EB has a wide range of variants, including Kindler syndrome, EB simplex, junctional EB, and dystrophic EB [1]. The overall incidence and prevalence of inherited EB were 19.60 and 8.22 per 1 million live births, respectively [2].

Patients present with mild to excessive fragility of the epithelium, resulting in blister formation and associated secondary lesions, such as erosions, ulcerations, crusts, and scars [3].

One of the major complications is the development of skin infections and cutaneous squamous cell carcinoma [4].

Total hip arthroplasty (THA) is performed to relieve pain, and most importantly, to improve the mobility of the hip joint. THA is known as one of the most effective performed surgical procedures in terms of patient satisfaction. Postoperative infection is the most common complication of THA, leading not only to severe delays in wound healing but also to significant financial burdens [5].

EB patients with active skin ulcers have an increased risk of skin infections [4]. One plausible explanation revolves around the administration of corticosteroids in the course of management. For this reason, the decision to undergo a THA might be questionable.

According to one case-controlled study, patients who were suffering from psoriatic skin disease and candidates for THA did not have any significant differences in terms of pain and disability compared to patients without psoriasis [6]. Since psoriasis is, as EB is, a dermatological condition, we felt more comfortable in proceeding with surgery.

Here we present a case of femoral head osteonecrosis in a patient with a history of EB that was successfully managed with THA without being complicated by postoperative infection.

## Case presentation

**Figure 1:**
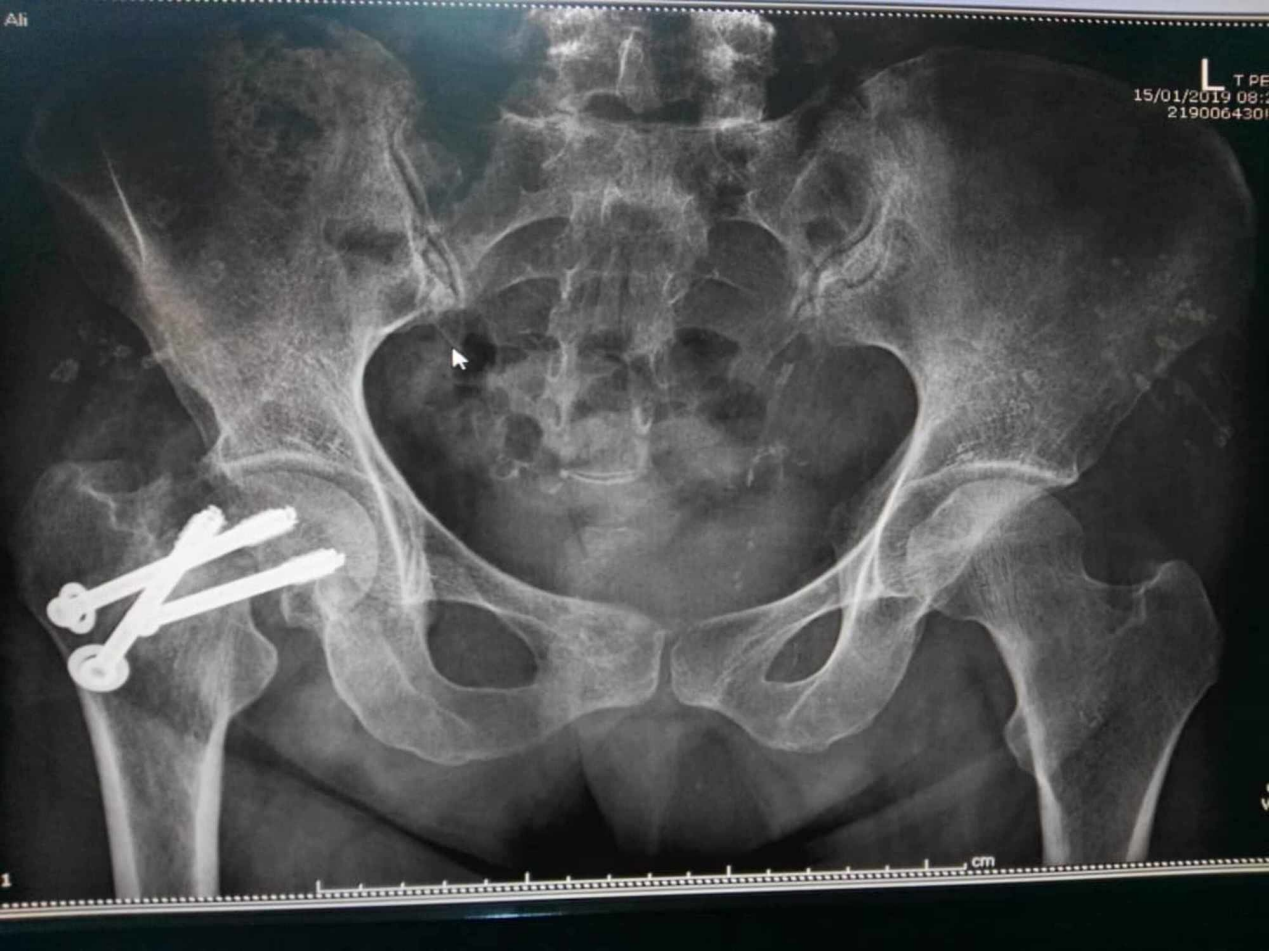
Open reduction and internal fixation of the right hip.

A 49-year-old female with a past medical history of EB, long-standing hypertension, and rheumatoid arthritis presented to the emergency department (ED) with right groin pain of one-hour duration after falling on her right side. After the fall, the patient was not able to bear weight and remained in a sitting position until she was taken to the ED. On the next day, she underwent a successful open reduction and internal fixation (ORIF) for her right hip without complications (Figure 1).

**Figure 2:**
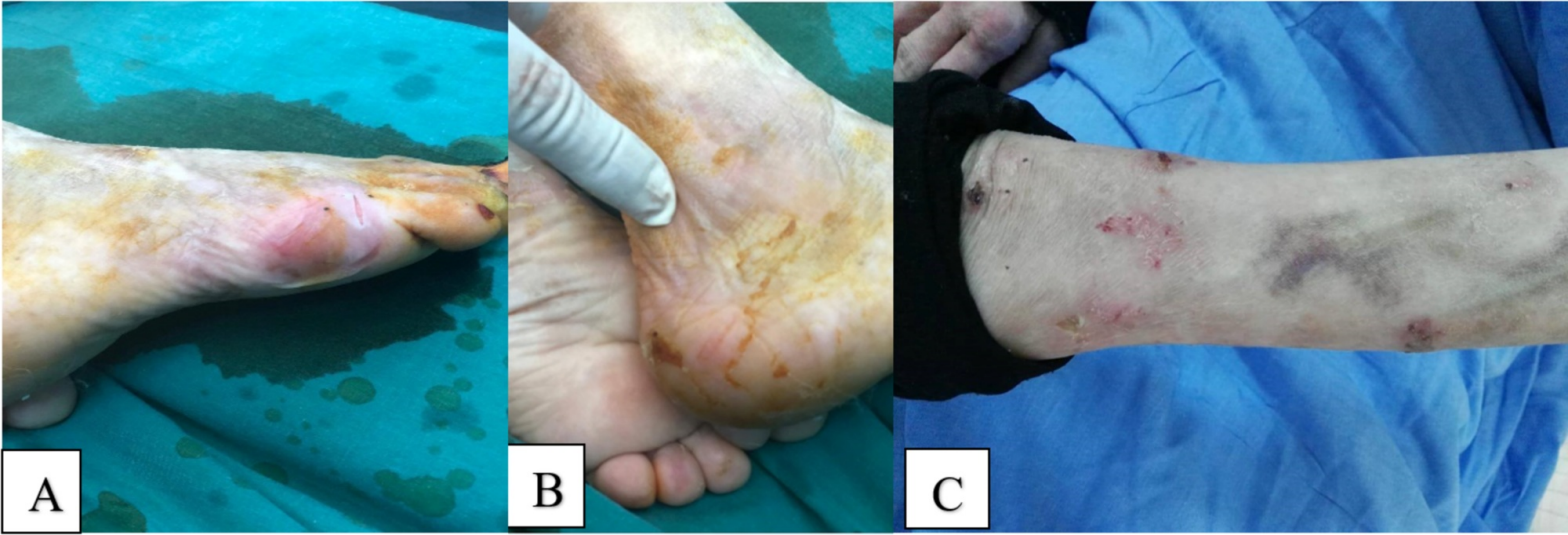
Skin manifestations of epidermolysis bullosa three months after open reduction and internal fixation. (A) The patient's right foot is shown here manifesting skin erosion. (B) The patient's right ankle is shown here manifesting fragile skin. (C) The patient's right forearm is shown here manifesting skin erosions.

Over the course of three months after the procedure, she experienced worsening of the pain accompanied by skin necrosis and total collapse of the femur head (Figure 2).

**Figure 3:**
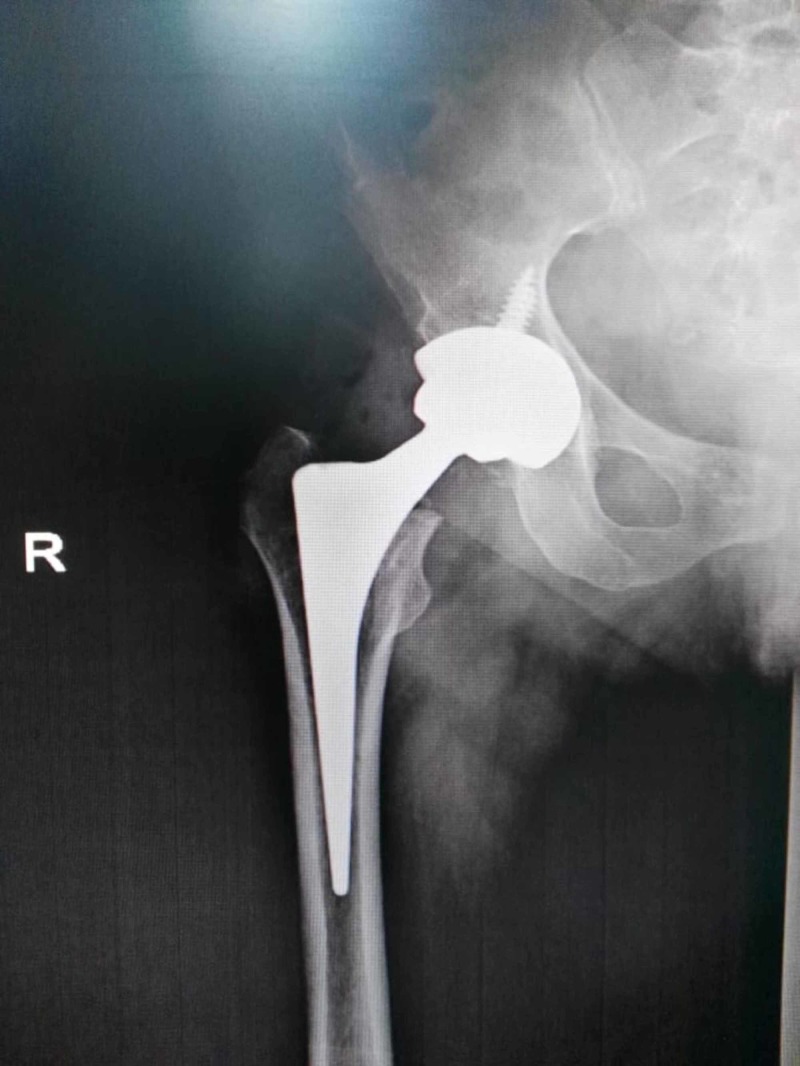
Total hip arthroplasty using a cementless (Zimmer) prosthesis and fixated via cannulated screws.

After that, the patient was scheduled to undergo a right THA. Before the surgery, a dermatology consult was obtained and approved the patient for surgery as long as there was careful and gentle handling of the skin during the application of povidone-iodine in the preoperative skin preparation. Furthermore, the dermatological team advised that only experts in anesthesia should proceed with intubation for surgery in order to avoid causing a mucosal injury in such a patient with fragile skin. A preoperative 2 g dose of the cefazolin IV was given along with the induction of anesthesia. The THA surgery was performed using a cementless (Zimmer, Warsaw, IN) prosthesis and fixated via cannulated screws (Figure 3).

The surgical wound was closed with a 2-0 nylon thread using a vertical mattress interrupted suture technique with gel, and no adhesive tape was used during wound closure. Vancomycin 500 mg IV and imipenem/cilastatin 500 mg IV were given for four days postoperatively. Postoperative pain control consisted of 10 mg morphine over six hours, 1 g of paracetamol given intravenously every six hours, and a 400-g dose of gabapentin given at bedtime. The pain score before the surgery was 10/10 and 5/10 immediately after the THA surgery. For a period of 25 days after the surgery, the patient did not complain of any pain. Upon follow-up, sutures were removed, and no surgical wound infection, rashes, or lacerations were noted.

## Discussion

Patients with EB can present with heterogeneous cutaneous manifestations. The manifestations can be classified into two major clinical subtypes: non-inflammatory (classical or mechanobullous) and inflammatory subtype, characterized by cutaneous inflammation [7].

Multidisciplinary care in EB patients is essential as this disease causes numerous complications such as skin fragility, blisters, and poor wound healing [8].

One of the major concerns with our patient is the fragility of the skin, which made us reluctant to perform THA. The benefits of THA have been more significant and have outweighed the risks of infection. The patient was experiencing severe hip pain which was expected to deteriorate more if left untreated, resulting in higher susceptibility to skin erosions. It was proven that early surgical intervention is recommended in deformities of bones or joints to prevent worsening damage over time [8].

Few cases of successful primary THA in methicillin-resistant Staphylococcus aureus (MRSA) carrier patients, who are at higher risk of infection, were documented in the literature which encouraged our decision to proceed [9].

Pressure sites are more exposed using the lateral or posterior approaches. Consequently, the anterior approach was chosen to avoid the associated complications, predominantly pressure ulcers [9].

Additionally, the direct anterior approach corresponds with decreased pain, fewer surgical complications, lower incidence of postoperative dislocation, and rapid recovery of patients [10].

Undergoing THA may end up in postoperative infection. One study shows that 1.08% of the patients who underwent primary and 2.1% of those who underwent revision arthroplasty had a postoperative infection. Only 0.45% of patients who underwent primary arthroplasty required revision for infection [11]. Given that our patient underwent ORIF one year ago, conversion THA was the choice to proceed with. Conversion THA according to the latest classification is considered as revision arthroplasty with increased risk of postoperative complications when compared to primary arthroplasty [12].

In general, orthopedic hardware infections are commonly due to Staphylococcus aureus or coagulase-negative staphylococci [13]. In terms of infection control, which is very essential in this particular case, the patient was given one dose of 2 g IV cefazolin prior to the surgery as prophylaxis. Surgical antimicrobial prophylaxis with cefazolin is advised for patients undergoing total hip replacement; acceptable alternatives include vancomycin or clindamycin [14]. Vancomycin is usually used as prophylactic antibiotic for patients known to be colonized with MRSA; otherwise cefazolin is the drug of choice [14]. The patient was not colonized with MRSA which led us to proceed with cefazolin. 

According to our hospital protocol, deep tissue culture was taken during the operation for assessment of infection. In order to reduce the risk of infection, vancomycin 500 mg IV and imipenem/cilastatin 500 mg IV were given for four days postoperatively. Fortunately, the culture result was negative for infection; therefore, no more antibiotics were given.

The fact that this patient is more susceptible to skin infection can not be ignored. One study has shown that pain, especially unresolved one, is considered one of the major obstacles to wound healing [15]. Adequate pain management will play a major role in this case. We made sure that our patient is experiencing the least possible pain by giving sufficient analgesics after the surgery. We used the following medications for pain management: 10 mg morphine over six hours, 1 g of paracetamol given intravenously every six hours, and a 400-g dose of gabapentin given at bedtime. 

## Conclusions

EB is a rare disorder that presents with excessive fragility and blistering of the skin. One of the major complications is the development of skin infections, which may hinder surgical intervention. We report the successful use of THA in a patient with EB at a high risk of infection. We encourage orthopedic surgeons dealing with patients suffering from dermatological conditions with fragile skin such as EB and decreased level of activity that requires THA to proceed with the surgical intervention after considering adequate infection control to improve quality of life. 
